# Multimodal Trajectory Prediction for Diverse Vehicle Types in Autonomous Driving with Heterogeneous Data and Physical Constraints

**DOI:** 10.3390/s24227323

**Published:** 2024-11-16

**Authors:** Maoning Ge, Kento Ohtani, Ming Ding, Yingjie Niu, Yuxiao Zhang, Kazuya Takeda

**Affiliations:** 1 Graduate School of Informatics, Nagoya University, Furo-cho, Chikusa-Ward, Nagoya 464-8601, Japan; 2 Zhejiang Fubang Technology Inc., Ningbo R&D Campus Block A, Ningbo 315048, China; 3 RoboSense Technology Co., Ltd., 701 Block B, 800 Naxian Road, Pudong, Shanghai 200131, China; 4 Tier IV Inc., Nagoya University Open Innovation Center, 1-3, Mei-eki 1-chome, Nakamura-Ward, Nagoya 450-6610, Japan

**Keywords:** multi-agent trajectory prediction, multimodal learning, Conditional Variational Autoencoder, Gaussian Mixture Model, autonomous driving

## Abstract

The accurate prediction of vehicle behavior is crucial for autonomous driving systems, impacting their safety and efficiency in complex urban environments. To address the challenge of multi-agent trajectory prediction, we propose a novel model integrating multiple input modalities, including historical trajectories, map data, vehicle features, and interaction information. Our approach employs a Conditional Variational Autoencoder (CVAE) framework with a decoder that predicts control actions using the Gaussian Mixture Model (GMM) and then converts these actions into dynamically feasible trajectories through a bicycle model. Evaluated on the nuScenes dataset, the model achieves great performance across key metrics, including minADE_5_ of 1.26 and minFDE_5_ of 2.85, demonstrating robust performance across various vehicle types and prediction horizons. These results indicate that integrating multiple data sources, physical models, and probabilistic methods significantly improves trajectory prediction accuracy and reliability for autonomous driving. Our approach generates diverse yet realistic predictions, capturing the multimodal nature of future outcomes while adhering to Physical Constraints and vehicle dynamics.

## 1. Introduction

The accurate prediction of vehicle behavior is crucial for autonomous driving systems, as it directly impacts their safety and efficiency [1,2,3]. By anticipating the future actions of surrounding road users, self-driving cars can effectively avoid collisions and navigate traffic smoothly. This capability is essential to establishing trust and integrating autonomous vehicles on public roads [4].

Humans exhibit an innate capacity for social cognition, or “theory of mind”, which allows them to comprehend and predict the intentions of others [5,6]. This enables them to anticipate the actions of those around them, facilitating smooth and successful navigation. Autonomous vehicles can draw inspiration from the way humans navigate complex social environments [7]. For autonomous systems to operate safely in real-world environments, they must demonstrate a level of social awareness and prediction similar to that of humans [8,9].

**Figure 1:**
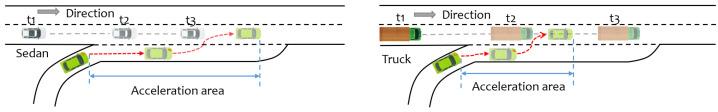
Trajectory prediction for lane merging maneuvers when merging in front of a sedan (**left**) and a truck (**right**), illustrating the predicted paths, acceleration areas, and decision-making process of the autonomous vehicle.

As illustrated in Figure 1, accurately predicting the behaviors of surrounding traffic, such as during overtaking maneuvers, plays a critical role in guiding the planning and decision-making of autonomous vehicles. By anticipating the actions of nearby vehicles like sedans and trucks, the system can safely and efficiently execute lane changes and other maneuvers. The differences in merging behavior between sedans and trucks are mainly due to their braking capability and size. Trucks have longer braking distances and larger physical dimensions, making it more challenging for them to slow down quickly and allow space for merging vehicles. In contrast, sedans are smaller and more agile, typically making it easier for the autonomous vehicle to merge safely and quickly. Given these differences, the ability to accurately predict multi-agent behavior has become critical for developing safe and efficient autonomous driving systems [10].

Current methods for multi-agent behavior prediction range from traditional kinematic models to advanced learning-based approaches [11,12]. Traditional methods, such as physics-based models, predict future positions using basic motion parameters such as velocity and acceleration [13,14,15]. Although straightforward, these models often fall short in complex and dynamic environments because they assume that agents move independently and neglect interaction dynamics. Rule-based models attempt to incorporate interaction heuristics, but their rigidity limits their adaptability to real-world scenarios [16,17].

Recent years have seen a shift toward learning-based models, which leverage data-driven techniques to capture complex patterns and interactions [11,12,18,19]. These approaches show promise in handling the intricacies of multi-agent scenarios. However, they still face significant challenges. Many current methods struggle to fully address real-world complexities, often overlooking crucial factors such as dynamic constraints, the ego agent’s motion, and rich environmental data from sources like high-definition maps and lidar sensors. Addressing these limitations is crucial for developing robust and reliable prediction systems capable of enhancing the safety and efficiency of autonomous driving in diverse real-world scenarios.

To better handle the uncertainty in vehicle behavior, many studies have explored probabilistic approaches for behavior prediction. For instance, Dynamic Bayesian Networks (DBNs) have been successfully applied in highway driving scenarios to predict vehicle maneuvers, demonstrating robustness and adaptability through empirical validation [20]. These DBN methods, which incorporate driver uncertainty and vehicle dynamics, have proven effective across various driving conditions [21]. Additionally, probabilistic architectures for long-term vehicle trajectory prediction are designed to manage uncertainties and provide flexible predictions in complex, multimodal traffic environments [22]. Recently, models such as the Conditional Variational Autoencoder (CVAE) [23,24,25,26,27] and Gaussian Mixture Model (GMM) [26,28] have shown potential in generating diverse and realistic trajectories, with CVAE producing varied possible outcomes from similar conditions and GMM capturing a range of trajectory patterns as a mixture of distributions. These probabilistic models underscore the importance of handling the inherent uncertainties and variability in multi-agent scenarios, offering a promising direction for improving prediction accuracy and safety in autonomous driving systems.

To address the complexity and uncertainty in multi-agent behavior prediction, this paper proposes a multimodal trajectory prediction model based on CVAE. The main contributions of this work are as follows:

1. The integration of heterogeneous data sources to improve prediction accuracy and diversity, generating trajectories for different vehicle types. The proposed model leverages various heterogeneous data sources, including high-definition maps, vehicle features, and interaction data between road agents, to generate customized trajectory predictions. By incorporating these contextual cues, the model captures the complex motion dynamics and interaction patterns of different road agents.
2. The proposed model uses a CVAE structure, with control variables as the predicted output, and represents the predicted trajectories using a GMM. This approach captures multiple plausible future trajectories and quantifies the uncertainty in the predictions, improving the model’s ability to handle complex traffic environments.
3. A bicycle model is incorporated as a Physical Constraint. The model introduces a bicycle model as a Physical Constraint in the learning-based framework for multi-agent trajectory prediction, ensuring the generated trajectories are physically feasible and realistic.

## 2. Related Works

### 2.1. Traditional Trajectory Prediction Methods

Traditional approaches to trajectory prediction have relied on simplified models and predefined rules, often failing to capture the complexity of real-world driving scenarios. These methods can be broadly categorized into two main types: physics-based models and rule-based approaches.

Physics-based models, including kinematic and dynamic models, predict future trajectories based on fundamental principles and observed motion parameters. They extrapolate future positions by considering current velocity, acceleration, and heading, assuming relatively constant motion patterns [13,14,15]. Although straightforward to implement, these models struggle to account for the unpredictable nature of human drivers and the influence of external factors such as road geometry and interactions with other vehicles.

Rule-based approaches utilize predefined heuristics to anticipate agent behavior [16,17]. These rules are often derived from traffic laws, common driving practices, or expert knowledge. However, the rigidity of these rules limits their ability to adapt to diverse and dynamic situations, especially when encountering unexpected maneuvers or complex interactions.

The inherent limitations of traditional methods stem from their inability to adequately model the complex factors influencing real-world driving behavior. They often neglect interaction dynamics, struggle with dynamic environments, and lack adaptability. These shortcomings underscore the need for more sophisticated models capable of learning from data and adapting to the complexities of real-world driving.

### 2.2. Learning-Based Trajectory Prediction Methods

In response to the limitations of traditional methods, learning-based approaches have gained significant traction in recent years. These methods leverage the power of machine learning to extract patterns and relationships from data, enabling them to handle the complexities of multi-agent trajectory prediction more effectively. Some prominent learning-based methods include Recurrent Neural Networks (RNNs), Generative Adversarial Networks (GANs), Conditional Variational Autoencoders (CVAEs), and Graph Neural Networks (GNNs).

RNNs excel at modeling sequential data, which makes them well suited for trajectory prediction [29,30,31,32,33,34,35,36]. By incorporating feedback loops, they can capture temporal dependencies in agent movements, learning how past positions influence future trajectories. This allows them to predict future states based on the historical movement patterns of agents.

GANs consist of two neural networks—a generator and a discriminator—trained in an adversarial manner [9,37,38,39]. The generator learns to produce realistic trajectories, while the discriminator aims to distinguish between real and generated trajectories. This adversarial training process pushes the generator to produce increasingly realistic and diverse trajectory predictions.

CVAEs are a type of variational autoencoder that conditions the encoding and decoding process on additional information, such as context or agent state. In trajectory prediction, they can model the inherent uncertainty and multi-modality of future trajectories by generating multiple possible outcomes given the same input conditions [23,24,25,26,27]. This probabilistic approach is especially useful in uncertain and dynamic driving environments.

GNNs are specifically designed to operate on graph-structured data, which makes them ideal for capturing interactions between agents [33,40,41,42]. By representing agents and their relationships as nodes and edges in a graph, they can learn complex interaction patterns and predict future trajectories based on the influence of neighboring agents.

The strengths of learning-based models include their ability to capture complex agent interactions and adapt to dynamic scenarios, improving accuracy in complex, real-world settings. However, these models face challenges such as computational complexity in training and deployment and the need for large, diverse datasets. They may also struggle to generalize to unfamiliar environments or scenarios different from their training data and often fall short of integrating real-world Physical Constraints or combining diverse data sources.

### 2.3. Incorporating Physical Constraints and Dynamics

While learning-based approaches have shown promise in multi-agent trajectory prediction, accurately modeling the physical world remains a significant challenge. Integrating Physical Constraints and dynamics, such as vehicle dynamics and road geometry, is crucial for developing realistic and reliable prediction models [43].

Recent research has explored the incorporation of vehicle dynamics into trajectory prediction frameworks [44,45]. These models consider factors like vehicle dimensions, steering angles, and tire slip to provide a more realistic representation of vehicle motion. By constraining predicted trajectories to physically plausible paths, these approaches aim to improve prediction accuracy and safety.

Studies have also highlighted the importance of considering vehicle-specific attributes, such as vehicle type (e.g., car, truck, motorcycle) and size, in prediction models [18]. Different vehicle types exhibit distinct motion characteristics and constraints, influencing their trajectory decisions. Incorporating this information can lead to more accurate and context-aware predictions [46,47].

## 3. Methodology

Our proposed model, MTP-HPC (Multimodal Trajectory Prediction with Heterogeneous Data and Physical Constraints), addresses the challenges of multi-agent trajectory prediction. The model integrates multiple data sources and advanced machine learning techniques to improve prediction accuracy for diverse vehicle types. The architecture of MTP-HPC consists of two main components: a feature extraction module and a trajectory generation module.

In the feature extraction stage, the model processes agent historical trajectories using Long Short-Term Memory (LSTM) to capture temporal dynamics. Simultaneously, vehicle features are processed through an embedding layer followed by a Multi-Layer Perceptron (MLP) to extract relevant features. To capture environmental context, Convolutional Neural Networks (CNNs) are employed to extract features from high-definition map data, which are then refined using fully connected (FC) layers. An attention-based interaction network models the interactions between various agents, such as vehicles and pedestrians. After feature extraction, the different data sources are concatenated into a unified 128-dimensional representation, forming the input for trajectory prediction.

In the trajectory generation stage, a CVAE framework is used to address the inherent uncertainty and multi-modal nature of future trajectories. The latent variable *z* is processed through an FC layer and then passed to the decoder. The decoder integrates a Gated Recurrent Unit (GRU), a GMM, and a kinematic bicycle model to produce realistic and diverse trajectory predictions. The GMM predicts control signals such as acceleration $a_{k}^{t}$ and steering angle $\delta_{k}^{t}$, which are used by the kinematic bicycle model to generate physically feasible trajectories. This approach ensures that the generated trajectories are diverse while adhering to environmental constraints and incorporating prediction uncertainty.

**Figure 2:**
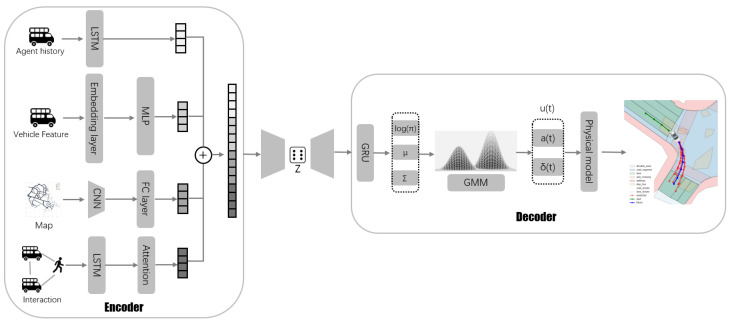
Architecture of the proposed MTP-HPC model, integrating historical trajectories, vehicle features, environmental data, and Physical Constraints to generate accurate and diverse future vehicle trajectories.

Figure 2 illustrates the complete architecture of our model, where each module collaborates to effectively capture the complex behavior of agents in multi-agent traffic scenarios.

### 3.1. Problem Definition

The multi-agent trajectory prediction task in autonomous driving scenarios involves forecasting the future trajectories of a dynamic set of interacting vehicles $A_{1},A_{2},\ldots ,A_{N_{t}}$, where $N_{t}$ represents the number of vehicles at time *t*, and each vehicle $A_{i}$ belongs to a specific semantic class $C_{i}$ (e.g., Car, Bus, Truck). Our goal is to predict their trajectories over the next *T* timesteps by considering their historical states.

At time *t*, we represent each vehicle $A_{i}$ using a comprehensive state vector $s_{i}^{t}\in R^{D}$ that includes kinematic states and external information. The external information incorporates vehicle-specific features, environmental features from HD maps, and interaction features.

The sequence of historical states over the past *H* timesteps is defined as

$$X=s_{i}^{t-H+1:t}=\left\{s_{i}^{t-H+1},s_{i}^{t-H+2},\ldots ,s_{i}^{t}\right\}$$

where X captures the complete movement history and contextual evolution of vehicle $A_{i}$ from the past *H* timesteps up to the current time *t*.

Our task is to predict the future states of each vehicle over the next *T* timesteps, represented as:

$$Y=r_{i}^{t+1:t+T}=\left\{r_{i}^{t+1},r_{i}^{t+2},\ldots ,r_{i}^{t+T}\right\}$$

While Y could, in principle, include predictions for all features such as position, velocity, and heading, in this work, we focus solely on predicting the position information $r_{i}^{t+T}$ of each vehicle.

Therefore, given the input variable X, our goal is to model the conditional probability distribution p(Y∣X) for the future positions Y of all vehicles. In this paper, this conditional probability distribution p(Y∣X) is represented by an associated probability density function.

This distribution captures how the future positions of all vehicles are influenced by their past movement history, vehicle-specific features, environmental context, and interactions with other neighboring vehicles. Through this modeling approach, we aim to comprehensively capture the dynamic behavior of the vehicles and their complex relationships with the surrounding environment, while specifically focusing on predicting accurate future positions.

### 3.2. Input Feature Extraction and Encoding

Accurate trajectory prediction relies on integrating multiple modalities to comprehensively understand vehicle behavior and environmental interactions. Our model combines various input modalities, including vehicle historical trajectories, vehicle attributes, map data, and interaction features, to capture key factors influencing vehicle dynamics and provide a rich context for accurate future trajectory predictions.

#### 3.2.1. Historical Trajectory Encoding

To capture temporal dependencies in the motion data, we use an LSTM network to encode the historical trajectory of each vehicle. For a scene at time *t* with $N_{t}$ vehicles, the input sequence for each vehicle *i* is defined as

$$X=s_{i}^{t-H+1:t}\in R^{H\times D}$$

where $s_{i}^{t-H+1:t}$ represents the sequence of historical states for vehicle *i* over the past *H* timesteps. Each state $s_{i}^{\tau }$ (for τ=t−H+1,…,t) includes features such as position, velocity, heading, and steering angle and has dimensionality *D*.

For each vehicle, we input its sequence of historical states into an LSTM network [31,48]. The LSTM processes each state in the sequence step-by-step, updating its hidden state at each time step to capture temporal dependencies in the vehicle’s motion. This iterative approach enables the LSTM to retain relevant motion patterns and dependencies within the sequence. The final hidden state vector, which is a 32-dimensional vector, serves as a summary of the vehicle’s motion history and is used as an input feature for the subsequent prediction model.

#### 3.2.2. Vehicle-Specific Features

To accurately capture the motion dynamics of different vehicles, our model integrates vehicle-specific features such as type and size. We classify vehicles into six types, car, bus, truck, trailer, construction vehicle, and emergency vehicle, each represented by an embedding layer that maps the type information into dense vectors [49]. This embedding captures latent relationships between different vehicle types, allowing the model to distinguish behavior patterns unique to each type.

For physical dimensions, we apply z-score normalization to the length, width, and height of vehicles. This normalization involves subtracting the mean and dividing by the standard deviation for each dimension, resulting in features that have a mean of zero and a standard deviation of one. This transformation reduces discrepancies in scale and enhances stability during model training.

The normalized dimensions are concatenated with the vehicle type embeddings to create a unified feature vector, which encodes both physical size and categorical information about the vehicle type. This vector is then fed into a multi-layer perceptron (MLP) [50] consisting of three fully connected layers with 128, 64, and 32 neurons, respectively. Each layer uses a ReLU activation function to introduce non-linearity, and the final output is a 32-dimensional embedding that encapsulates the key characteristics of each vehicle.

We train the MLP from scratch on our dataset using the Adam optimizer, enabling it to learn complex interactions between vehicle types and dimensions. The resulting embedding captures key characteristics of each vehicle, enhancing the trajectory prediction model’s ability to generate precise, context-sensitive predictions.

#### 3.2.3. Map Feature Extraction

To incorporate environmental context into the trajectory prediction model, we use a local semantic map centered on each vehicle. The input map, $M_{i}^{t}$, is a binary tensor where each element can be 0 or 1, with dimensions h×w×l, where *h* and *w* represent height and width, and *l* indicates the number of semantic layers. Each layer corresponds to features such as the drivable area, road divider, lane divider, stop line, and pedestrian crossing.

**Figure 3:**
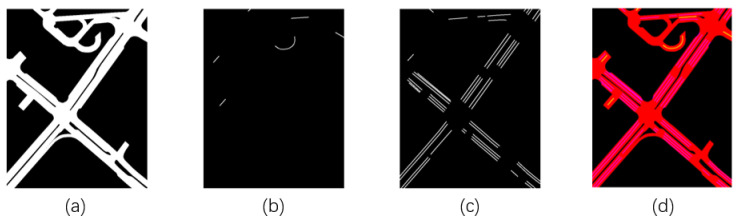
The semantic map includes three separate channels: (**a**) drivable areas, (**b**) road dividers, (**c**) lane dividers, and (**d**) a composite RGB image combining the three channels.

As shown in Figure 3, the semantic map includes three separate channels: drivable areas, road dividers, and lane dividers. Additionally, a composite RGB image combines these channels to provide a comprehensive view of the local road environment around the vehicle.

To ensure spatial consistency, the map is aligned with the vehicle’s heading direction. After alignment, the map patch is processed using a CNN [51] with multiple convolutional layers, which capture both broad spatial patterns and finer details.

**Figure 4:**
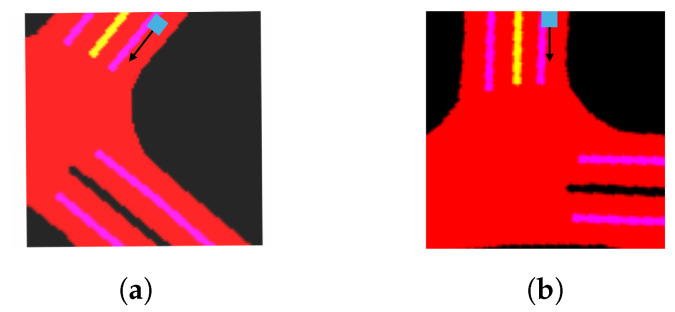
Comparison of mask maps before and after rotation based on the vehicle’s heading direction. (**a**) Before rotation. (**b**) After rotation.

To align the local semantic map with the vehicle’s heading direction, the map is rotated to match the orientation of the vehicle’s travel. In Figure 4, the left image (a) shows the original map before rotation, and the right image (b) illustrates the map after rotation. This alignment ensures spatial consistency, which is crucial for accurate trajectory prediction. The cyan square represents the vehicle, and the black arrow indicates its heading direction.

The resulting feature representation is a 32-dimensional vector that encapsulates key spatial information from the vehicle’s local environment. This vector, combined with the vehicle’s motion history, enables the model to generate more precise and context-aware trajectory predictions, taking into account both dynamic behavior and static road infrastructure.

Additionally, this feature extraction process can be extended to include other sensor data, such as LIDAR or camera images, further enhancing the model’s understanding of complex traffic scenarios.

#### 3.2.4. Interaction Network

To model the interaction of vehicles with other agents in the environment, we represent the scene as a graph, where each agent is treated as a vertex, and edges indicate potential interactions [26]. Each vertex is associated with a semantic category, such as “Car”, “Bus”, or “Pedestrian”. A directed edge from entity $E_{i}$ to $E_{j}$ exists if $E_{i}$ can influence $E_{j}$. This potential influence is determined by evaluating the distance between their positions, represented as

$$∥p_{i}-p_{j}∥\;\leq \;d_{\mathrm{interaction}}$$

where $p_{i}$ and $p_{j}$ are the 2D coordinates of entities $E_{i}$ and $E_{j}$, and $d_{\mathrm{interaction}}$ is a threshold distance based on the interaction type between entities $E_{i}$ and $E_{j}$ (e.g., “car-pedestrian”, “car-car”).

The interaction network represents the relative relationship between the current agent and its neighboring agents using edge features. For the same type of edge (e.g., “car-car”), the model aggregates these edge features using element-wise summation. This aggregation method enables the model to flexibly handle varying numbers of neighboring entities while preserving the interaction information conveyed by each edge type. The aggregated edge features for each type are then fed into an LSTM with shared weights across all connections of the same type (e.g., “car-pedestrian”), which encodes the influence exerted by neighboring entities over time.

To capture the combined influence of different types of edges on the target entity, the model employs an additive attention mechanism [52]. This mechanism assigns dynamic weights to each edge type, allowing the model to emphasize the most relevant interactions for the target agent within a given context. Specifically, the attention score $e_{ij}$ between the target agent’s state $q_{i}$ and each edge type $k_{j}$ is calculated as follows:

$$e_{ij}=v^{⊤}\tan h\left(W_{q}q_{i}+W_{k}k_{j}\right)$$

where $q_{i}$ is the encoded feature of the target agent, $k_{j}$ represents the LSTM-encoded feature for each edge type, and $W_{q}$, $W_{k}$, and *v* are learnable parameters. The scores are then normalized with a Softmax function to produce attention weights $\alpha_{ij}$:

$$\alpha_{ij}=\frac{\exp(e_{ij})}{\sum_{j^{\prime }=1}^{M}\exp\left(e_{ij^{\prime }}\right)}$$

where *M* is the total number of neighbor types. Finally, the model computes a weighted sum of the edge-type encodings, yielding the final influence representation for the target agent:

$$\mathrm{Influence}=\sum_{j=1}^{M}\alpha_{ij}k_{j}$$

This attention mechanism ensures that the model focuses on the most significant interactions, producing a 32-dimensional weighted representation of the overall influence that neighboring agents exert on the target agent. By dynamically adjusting these weights based on specific interactions, the model gains a comprehensive understanding of the relationships among multiple entities, enhancing its performance in complex, multi-agent scenarios.

### 3.3. Prediction Module

The prediction module is used to forecast future trajectories of multiple interacting agents in complex driving environments. It consists of two main components: Latent Variable Modeling and a Decoder with Physical Constraints. In the following sections, we provide a detailed explanation of each component and its role in generating robust and context-aware predictions.

#### 3.3.1. Latent Variable Modeling

To effectively capture the multimodal nature of future trajectories, the prediction module employs an Information-Maximizing Variational Autoencoder (infoVAE) framework [53]. Additionally, we introduce a high-level latent variable *z*, which represents a finite set of possible high-level behaviors [26]. The latent variable *z* encodes these behaviors, enabling the model to represent multiple possible future outcomes. The overall conditional probability distribution of future trajectories p(Y∣X) is expressed by marginalizing over the latent variable:

$$p\left(Y|X\right)=\sum_{z}p_{\theta_{2}}\left(Y|X,z\right)\;p_{\theta_{1}}\left(z|X\right)$$

where $X\in R^{n}$ is the encoded context vector, including historical trajectory, vehicle features, interaction features, and environmental information, with n=128; $Y\in R^{m}$ represents the predicted future trajectory, with m=2; and *z* is the set of discrete latent variables, consisting of 20 discrete elements. Here, $p_{\theta_{1}}\left(z|X\right)$ and $p_{\theta_{2}}\left(Y|X,z\right)$ are parameterized by $\theta_{1}$ and $\theta_{2}$, respectively, representing conditional probability distributions.

**Training Loss Function**

In the infoVAE framework [53], a mutual information (MI) term is introduced in the loss function by maximizing the correlation between the latent variable *z* and the input context x. The MI term ensures that the latent variable *z* effectively captures the diverse modalities of future behavior given the context.

The objective of training the model is to minimize the following loss function:

$$L=-\lambda \cdot D_{\mathrm{KL}}\left(q_{\phi }\left(z|X,Y\right)\|p_{\theta_{1}}\left(z|X\right)\right)-E_{q_{\phi }\left(z|X,Y\right)}\left[\log p_{\theta_{2}}\left(Y|X,z\right)\right]+\alpha \cdot I\left(X;z\right)$$

where the first term $-\lambda \cdot D_{\mathrm{KL}}\left(q_{\phi }\left(z|X,Y\right)\|p_{\theta_{1}}\left(z|X\right)\right)$ is the Kullback–Leibler divergence term, which regularizes the posterior distribution to be close to the prior distribution, preventing overfitting and encouraging smoothness in the latent space; the second term $-E_{q_{\phi }\left(z|X,Y\right)}\left[\log p_{\theta_{2}}\left(Y|X,z\right)\right]$ is the reconstruction loss, ensuring that the predicted trajectory is close to the ground truth trajectory; and the third term α·I(X;z) is the mutual information term, which maximizes the dependency between the context x and the latent variable *z*.

The mutual information I(X;z) [54] is defined as

$$I\left(X;z\right)=E_{p_{\theta_{1}}\left(X,z\right)}\left[\log\frac{p_{\theta_{1}}\left(z|X\right)}{p_{\theta_{1}}\left(z\right)}\right]$$

where the unconditional latent distribution $p_{\theta_{1}}\left(z\right)$ is obtained by averaging over all x in the batch.

**Model Training**

During model training, the encoder receives the input information x and the corresponding ground truth future trajectory y, generating the posterior distribution $q_{\phi }\left(z|X,Y\right)$ [55]. The encoder and decoder networks are optimized to minimize the total loss function L. The inclusion of the mutual information term increases the interpretability and robustness of the latent representations, enabling the model to generate diverse and contextually appropriate trajectories for each agent. By balancing reconstruction accuracy, latent space regularization, and informativeness of the latent variables, the infoVAE framework generates a comprehensive set of possible future outcomes given the current context.

#### 3.3.2. Decoder with Physical Constraints

The decoder generates future trajectories that are diverse and dynamically feasible by incorporating vehicle kinematic constraints. The prediction process involves three main components, a GRU module, a bivariate Gaussian distribution, and a bicycle model, which work together in sequence to generate physically feasible trajectories.

A. GRU Module for State Prediction

The first component of the decoder is a GRU [56] module that processes the encoded information. The GRU module predicts a sequence of control actions over the specified prediction horizon. It takes as input the latent variable *z* sampled from the latent space and the encoded context vector x. At each time step *t*, the GRU outputs the parameters of a bivariate Gaussian distribution for control actions $u^{t}=\left[a_{k}^{t},\delta_{k}^{t}\right]$, where $a_{k}^{t}$ is the acceleration and $\delta_{k}^{t}$ is the steering angle. These parameters are then used to define the distribution of possible control actions.

B. Bivariate Gaussian Distribution for Control Modeling

To model the uncertainty in the GRU’s predictions, we employ a bivariate Gaussian distribution to capture the joint distribution of possible control actions. This approach allows us to represent the variability in acceleration and steering angle across future time steps, providing a probabilistic framework that enables the generation of diverse and feasible trajectories.

In this context, a Gaussian distribution refers to a probability distribution that is fully described by its mean and covariance matrix. For each time step *t*, the GRU outputs parameters for a bivariate Gaussian distribution that defines the probability distribution of control action variables U [57]. The probability distribution of control actions at each time step *t* is given by

$$p\left(U|X,z\right)=N\left(u^{t}|\mu^{t},\Sigma_{u}^{t}\right)$$

where $\mu^{t}=\left[\mu_{a}^{t},\mu_{\delta }^{t}\right]$ is the mean vector, representing the expected values of acceleration $a_{k}^{t}$ and steering angle $\delta_{k}^{t}$, and $\Sigma_{u}^{t}$ is the covariance matrix that captures the uncertainties and correlations between these control actions:

$$\Sigma_{u}^{t}=\left[\begin{array}{cc} {\left(\sigma_{a}^{t}\right)}^{2} & \rho_{a\delta }^{t}\;\sigma_{a}^{t}\;\sigma_{\delta }^{t}\\ \rho_{a\delta }^{t}\;\sigma_{a}^{t}\;\sigma_{\delta }^{t} & {\left(\sigma_{\delta }^{t}\right)}^{2} \end{array}\right]$$

Here, ${\left(\sigma_{a}^{t}\right)}^{2}$ and ${\left(\sigma_{\delta }^{t}\right)}^{2}$ denote the variances of acceleration and steering angle at time *t*, respectively, while $\rho_{a\delta }^{t}$ represents the correlation between them. This covariance structure captures the range of possible control actions and their interdependencies, allowing for a flexible representation of uncertainties in control at each step.

By using this probabilistic model, we can sample control actions $u^{t}$ from this distribution at each time step. These sampled actions provide a range of dynamically feasible trajectories when combined with the Physical Constraints imposed by the kinematic model.

C. Bicycle Model for Trajectory Generation

Finally, to ensure physical feasibility, the control actions sampled from this bivariate Gaussian distribution are integrated with the vehicle’s kinematic model. We employ the bicycle model [58], a simplified representation of vehicle dynamics, to transform these control actions into vehicle trajectories in the position space. The equations governing the bicycle model are given as follows:

$${\dot{x}}_{k}^{t}=v^{t}\cos\left(\theta_{k}^{t}\right)$$

$${\dot{y}}_{k}^{t}=v^{t}\sin\left(\theta_{k}^{t}\right)$$

$${\dot{\theta_{k}}}^{t}=\frac{v_{k}^{t}}{L}\tan\left(\delta_{k}^{t}\right)$$

$${\dot{v}}_{k}^{t}=a_{k}^{t}$$

where $\left(x_{k}^{t},y_{k}^{t}\right)$ are the coordinates of the vehicle in the 2D plane at time *t*, $\theta_{k}^{t}$ is the vehicle’s heading angle, $v_{k}^{t}$ is the vehicle’s speed, $\delta_{k}^{t}$ is the steering angle, $a_{k}^{t}$ is the acceleration, and *L* is the wheelbase of the vehicle. These equations describe the evolution of the vehicle’s position $\left(x_{k}^{t},y_{k}^{t}\right)$ and the rate of change in heading over time under the influence of the control actions $a_{k}^{t}$ and $\delta_{k}^{t}$.

## 4. Experiments

### 4.1. Datasets

For this work, we utilize the nuScenes dataset [59], a large-scale dataset designed for autonomous driving research. nuScenes provides a comprehensive set of multi-modal sensor data, high-definition maps, and detailed annotations, making it an ideal resource for trajectory prediction tasks.

The dataset comprises 1000 scenes, each 20 s long, recorded in diverse urban environments across Boston and Singapore. The data are captured at a frequency of 2 Hz using multiple sensors, including LiDAR, radars, and cameras, providing synchronized, high-resolution information about the environment. Annotations are available for various types of dynamic agents (e.g., cars, buses, trucks, and pedestrians) and static elements such as road infrastructure.

In addition to the dynamic agent data, nuScenes offers high-definition semantic maps, which include detailed information (e.g., lane boundaries, drivable areas, road dividers, and crosswalks). These maps are integral to understanding the environment in which the agents operate and are crucial for tasks involving trajectory prediction in complex urban scenarios.

For our trajectory prediction task, we extract historical trajectories up to 2 s as input features, along with corresponding local map patches. The future trajectories of 6 s serve as ground truth for training and evaluation. We adhere to the official nuScenes data split, using 700 scenes for training, 150 for validation, and 150 for testing.

### 4.2. Implementation Details

The model was implemented using PyTorch and trained on an NVIDIA RTX A6000 GPU with 48 GB of memory for 20 epochs. A batch size of 512 was used with the Adam optimizer and an initial learning rate of 0.002, decaying exponentially by 0.9999 per epoch. Gradient clipping with a cap of 1.0 was applied to ensure training stability.

The dataset was preprocessed by transforming agent trajectories into a local coordinate system, setting each agent’s initial position at the origin and aligning their heading.

The model employed a CVAE structure, which incorporated both KL divergence and MI terms. The KL weight started at 0 and gradually increased to 100 using a sigmoid schedule, allowing the model to prioritize reconstruction early in training and later focus on regularizing the latent space. The MI term was fixed at α=1, ensuring the latent variable *z* captured relevant information from the input x, balancing multimodality and latent space structure.

The model was evaluated on 150 test scenes from the nuScenes dataset. Multiple plausible trajectories were generated by sampling from GMM, accounting for uncertainty in agent behavior and ensuring realistic trajectory predictions.

For a more detailed discussion on the model’s runtime performance during online inference, including metrics such as inference time, frame rate, and memory usage, please refer to Appendix A.

### 4.3. Evaluation Metrics

To evaluate the performance of the proposed trajectory prediction model, as in previous work [26,27,32,33,34,60,61,62], we used the following key metrics:

Minimum Average Displacement Error (minADE)

minADE measures the minimum average Euclidean distance between the predicted trajectory and the ground truth trajectory across all time steps among the predicted trajectories. It primarily evaluates the prediction accuracy of the model across the entire trajectory:

$${\mathrm{minADE}}_{k}=\min_{i=1,\ldots ,k}\left(\frac{1}{T}\sum_{t=1}^{T}{∥y_{t}^{i,\mathrm{pred}}-y_{t}^{\mathrm{true}}∥}_{2}\right)$$

where *i* is the index of a sampled predicted trajectory from the set of generated trajectories, and *T* is the prediction horizon. Here, ${∥\cdot ∥}_{2}$ denotes the Euclidean (L2) norm, which is used to measure the Euclidean distance between the predicted and true trajectories.

Minimum Final Displacement Error (minFDE)

minFDE measures the minimum Euclidean distance between the predicted and true positions at the final time step among the predicted trajectories. This metric focuses on the final prediction accuracy, especially at the last predicted point:

$${\mathrm{minFDE}}_{k}=\min_{i=1,\ldots ,k}\left({∥y_{T}^{i,\mathrm{pred}}-y_{T}^{\mathrm{true}}∥}_{2}\right)$$

where *i* refers to the index of the closest predicted trajectory from the set.

Miss Rate at Distance *d* (${\mathrm{MissRate}}_{k,d}$)

${\mathrm{MissRate}}_{k,d}$ measures the proportion of predicted trajectories where the maximum pointwise L2 distance exceeds a threshold *d*. If any time step’s error is greater than *d*, the trajectory is considered a miss. This metric evaluates the model’s ability to predict trajectories within a certain tolerance, assessing its prediction accuracy. It is defined as

$${\mathrm{MissRate}}_{k,d}=\frac{1}{N}\sum_{i=1}^{N}I\left[\min_{j=1,\ldots ,k}\left(\max_{t=1,\ldots ,T}{∥y_{t}^{j,\mathrm{pred}}-y_{t}^{\mathrm{true}}∥}_{2}\right)>d\right]$$

where *k* refers to the top *k* predicted trajectories, *d* is the threshold, and *N* is the total number of agents. In our case, d=2 m, a threshold commonly used in the nuScenes benchmark for trajectory prediction tasks. This value is chosen to reflect a balance between precision and safety in autonomous driving, where predicting within a 2 m margin is crucial for maintaining safe distances from other vehicles and obstacles in complex driving environments.

Off-Road Rate

Off-Road Rate evaluates the percentage of predicted trajectories that leave the drivable area. This metric is used to assess the model’s adherence to driving constraints, ensuring that the predicted trajectories stay within the drivable area:

$$\mathrm{Off}\text{-}\mathrm{Road}\;\mathrm{Rate}=\frac{\mathrm{Number}\;\mathrm{of}\;\mathrm{off}\text{-}\mathrm{road}\;\mathrm{trajectories}}{\mathrm{Total}\;\mathrm{number}\;\mathrm{of}\;\mathrm{trajectories}}$$

Kernel Density Estimate Negative Log-Likelihood (KDE NLL)

KDE NLL measures the quality of the predicted probability distribution by evaluating the likelihood of the ground truth trajectory under a kernel density estimate formed from the predicted trajectories. This metric captures the overall quality of the model’s multimodal behavior and uncertainty:

$$\mathrm{KDE}\;{\mathrm{NLL}}_{k}=-\frac{1}{N}\sum_{i=1}^{N}\log\left(\frac{1}{K}\sum_{k=1}^{K}N\left(y_{t}^{\mathrm{true}}|y_{t}^{k,\mathrm{pred}},\Sigma_{p}\right)\right)$$

where $N(\cdot |y_{t}^{k,\mathrm{pred}},\Sigma_{p})$ represents a Gaussian distribution centered at the predicted point $y_{t}^{k,\mathrm{pred}}$ with covariance $\Sigma_{p}$, *K* is the number of predicted trajectories, and *N* is the number of agents.

These metrics comprehensively evaluate the model’s prediction accuracy, multimodal behavior coverage, and adherence to driving constraints, providing a thorough assessment of its performance in real-world scenarios.

### 4.4. Results and Analysis

#### 4.4.1. Quantitative Results

We evaluate our proposed method against several state-of-the-art baseline models on the nuScenes dataset. Table 1 presents a comprehensive comparison of our method with these baselines across various metrics. The subscripts 1,5,10 in MinADE, MinFDE, and MissRate indicate that the metric is calculated by selecting the best trajectory from the top 1, 5, or 10 predicted trajectories.

Our proposed MTP-HPC method demonstrates strong performance on all evaluation metrics. For single-trajectory prediction, our method achieves a MinADE_1_ of 2.14 and a MinFDE_1_ of 5.03, indicating high accuracy in predicting the most probable future path of vehicles.

In multiple trajectory prediction scenarios, our method also shows robust performance with MinADE_5_ and MinADE_10_ values of 1.26 and 0.99, respectively. Notably, our method achieves the best performance in MinFDE_5_ (2.85) and MinFDE_10_ (2.16) among all compared methods, highlighting its effectiveness in long-term trajectory prediction.

The low ${\mathrm{MissRate}}_{5,2}$ of 0.41 demonstrates our model’s ability to generate accurate predictions within a 2 m threshold, which is crucial for safety in autonomous driving applications. Furthermore, our model achieves a low Off-Road Rate of 0.02, showcasing its effectiveness in predicting trajectories that adhere to road constraints.

**Figure 5:**
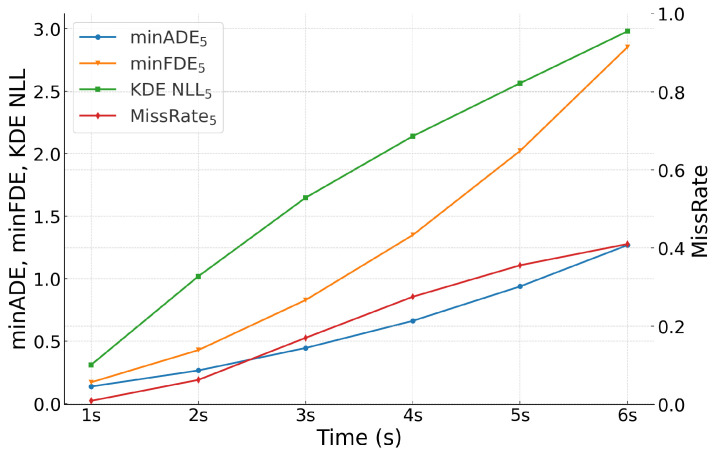
Trajectory prediction metrics over different prediction horizons for all vehicles.

To further analyze our model’s performance, we examine how different metrics change over various prediction horizons. Figure 5 illustrates the average values from 1 to 6 s.

As expected, prediction errors generally increase with longer time horizons. The minADE_5_ and minFDE_5_ metrics show a steady increase, reflecting the growing uncertainty in predicting vehicle positions further into the future. The KDE NLL_5_ metric also increases over time, indicating that the model’s probabilistic predictions become less certain for longer-term forecasts.

**Figure 6:**
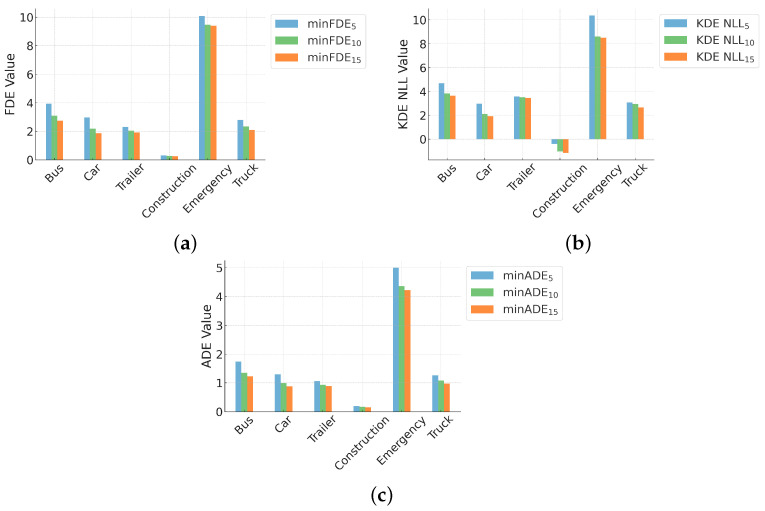
Performance metrics across vehicle types for varying numbers of predicted trajectories: (**a**) minFDE, (**b**) KDE NLL, and (**c**) minADE for 5, 10, and 15 predictions.

To evaluate the versatility of our model, we analyze its performance across different vehicle types. Table 2 presents the minADE_5_, minFDE_5_, and KDE NLL _5_ metrics for various vehicle types at 2, 4, and 6 s prediction horizons. Figure 6 illustrates the performance metrics for different vehicle types when considering 5, 10, and 15 predicted trajectories.

Our model demonstrates good performance across the majority of vehicle types. Analysis results show that the model can consistently generate accurate trajectory predictions for all evaluated vehicle types.

Notably, construction vehicles exhibit the lowest error rates across all metrics and time horizons. On the other hand, emergency vehicles show relatively higher error rates, primarily due to the limited number of samples for this vehicle type in the dataset.

For vehicle types that occupy the majority of road traffic, such as cars, trucks, buses, and trailers, our model demonstrates stable and reliable prediction capabilities. These results highlight the applicability and effectiveness of our method in real-world traffic scenarios.

The KDE NLL metric further confirms the excellent performance of our model across different vehicle types, with most vehicle types achieving low KDE NLL values. This suggests that our model produces accurate and reliable probability distributions for trajectory predictions across various vehicle categories.

#### 4.4.2. Qualitative Analysis

**Figure 7:**
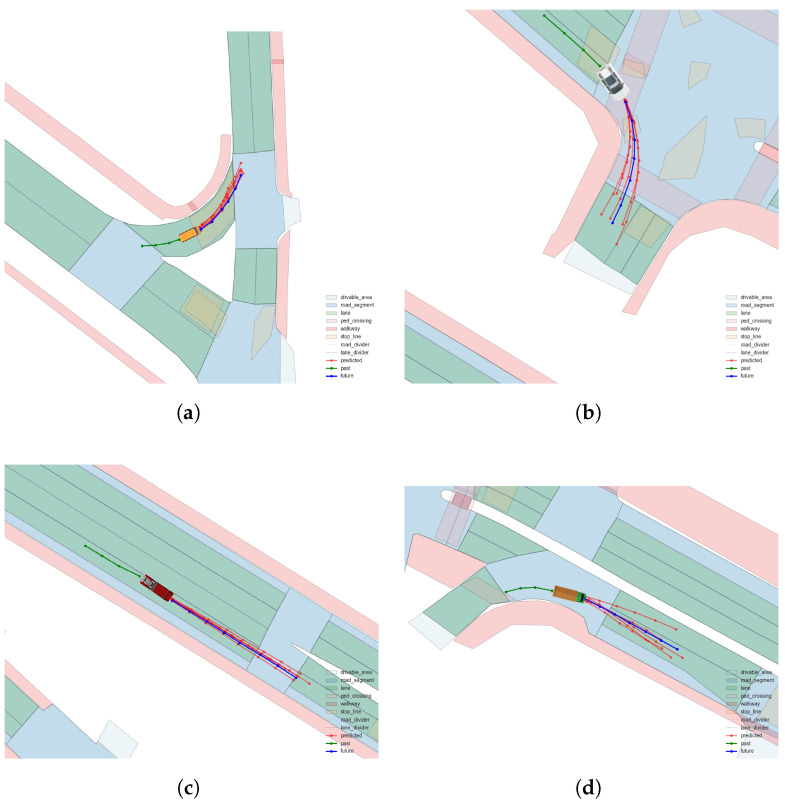
Trajectories of different vehicle types. (**a**) Bus trajectory. (**b**) Car trajectory. (**c**) Trailer trajectory. (**d**) Truck trajectory.

To visually evaluate our model’s performance, we conducted a visualization analysis of trajectory predictions for different vehicle types. Figure 7 shows the predicted trajectories for four typical vehicle types: bus, car, trailer, and truck.

From the figure, we can observe that our model is capable of generating diverse trajectory predictions for different types of vehicles. The predicted trajectories for each vehicle type exhibit a range of distributions, reflecting the model’s multiple predictions for possible future paths.

These visualization results demonstrate the consistency of our model in handling different vehicle types. Whether for large vehicles (such as buses and trailers) or smaller vehicles (like cars), the model is able to generate a series of possible trajectories. The distribution of predicted trajectories reflects the model’s estimation of uncertainty in future positions.

#### 4.4.3. Ablation Studies

To understand the contribution of each component in our model, we conducted a series of ablation studies. Table 3 presents the results of these studies, showing how different combinations of components affect the model’s performance.

The ablation studies investigate the impact of three key components: map information, Physical Constraints, and vehicle features. From the results, we can observe that the base model, without any additional components, shows the highest error rates across all metrics. Each component, when added individually to the base model, improves performance.

The combination of all three components yields the best performance across all metrics, with a minADE_5_ of 1.26, a minFDE_5_ of 2.85, and a KDE NLL_5_ of 2.89. The Physical Constraint and vehicle feature components, when combined with map information, show synergistic effects, further reducing error rates.

These results demonstrate the importance of each component in our model architecture. The map information provides crucial context about the environment, the Physical Constraints ensure realistic predictions, and the vehicle features allow the model to adapt to different vehicle types. The synergistic effect of these components results in a model that outperforms simpler variants across all evaluated metrics.

## 5. Conclusions

In this work, we proposed MTP-HPC, a novel multi-agent trajectory prediction model that effectively integrates multiple input modalities, including historical trajectory data, interaction features, and environmental context. By leveraging a CVAE framework along with a physically constrained decoder, our model captures the multimodal nature of future outcomes, generating diverse yet dynamically feasible trajectory predictions. This multimodal integration enables the model to handle complex real-world driving environments, accurately predicting future trajectories for various vehicle types, such as cars, trucks, and emergency vehicles.

The performance evaluation on the nuScenes dataset demonstrates significant improvements in both accuracy and realism. Our model achieves great results in key metrics such as minADE and minFDE, reflecting its ability to generate precise short- and long-term predictions. The low Off-Road Rate further underscores the model’s effectiveness in producing safe and realistic trajectories that comply with road constraints. Additionally, the competitive KDE NLL scores highlight the model’s ability to manage uncertainty and generate multiple plausible future paths, which is crucial for dynamic urban driving scenarios.

Despite these strong results, there are areas for future improvement. Currently, our model uses a CNN architecture for map feature processing and interaction modeling due to its stability in spatial feature extraction and computational efficiency. In future work, we plan to explore more recent network architectures, such as Vision Transformers and Graph Transformer Networks, aiming to further improve model performance while balancing prediction accuracy and computational efficiency. Additionally, as prediction time horizons increase, our model’s prediction error also grows, indicating the need for better methods to capture long-term dependencies. Accurate long-term predictions are essential for decision-making in autonomous driving. Furthermore, the model’s higher error rates for rare vehicle types, such as emergency vehicles, suggest the need for further refinement through targeted data augmentation or specialized training to improve generalizability across diverse agent types.

In future work, we plan to evaluate the model on additional datasets to further assess its generalization capabilities across different environments and driving conditions. We will also explore online deployment, enabling the model to operate in real time and continuously adapt to evolving traffic scenarios.

## Figures and Tables

**Table 1:** Comparison with baseline models on the nuScenes dataset (units: meters for minADE, minFDE; percentage for MissRate and Off-Road Rate).

	MinADE_1_ (m)	MinADE_5_ (m)	MinADE_10_ (m)	MinFDE_1_ (m)	MinFDE_5_ (m)	MinFDE_10_ (m)	${\mathrm{MissRate}}_{5,2}$ (%)	${\mathrm{MissRate}}_{10,2}$ (%)	Off-Road Rate (%)
Const vel and yaw	4.61	4.61	4.61	11.21	11.21	11.21	91	91	14
Physics oracle	3.69	3.69	3.69	9.06	9.06	9.06	88	88	12
MTP [60]	4.42	2.22	1.74	10.36	4.83	3.54	74	67	25
Multipath [61]	4.43	1.78	1.55	10.16	3.62	2.93	78	76	36
CoverNet [62]	-	2.62	1.92	11.36	-	-	76	64	13
Trajectron++ [26]	-	1.88	1.51	9.52	-	-	70	57	25
MHA-JAM [32]	3.77	1.85	1.24	8.65	3.85	2.23	59	45	7
PGP [33]	-	1.27	0.94	7.17	-	-	52	34	3
FRM [27]	-	1.18	**0.88**	6.59	-	-	48	30	2
CASPNet++ [34]	2.74	**1.18**	0.93	6.19	-	-	50	**30**	**1**
Our Method (MTP-HPC)	**2.14**	1.26	0.99	**5.03**	**2.85**	**2.16**	**41**	32	2

**Table 2:** Trajectory prediction performance across vehicle types and time horizons.

Type	minADE_5_			minFDE_5_			KDE NLL_5_		
	2 s	4 s	6 s	2 s	4 s	6 s	2 s	4 s	6 s
BUS	0.23	0.79	1.74	0.31	1.58	3.94	1.29	3.25	4.70
CAR	0.18	0.60	1.30	0.23	1.18	2.98	0.17	1.82	2.98
TRAILER	0.17	0.54	1.07	0.22	1.00	2.31	−0.10	2.30	3.59
CONSTRUCTION	0.05	0.13	0.20	0.06	0.19	0.32	−2.66	−1.09	−0.40
EMERGENCY	0.51	2.58	5.00	0.69	5.26	10.09	4.47	8.52	10.38
TRUCK	0.18	0.59	1.26	0.24	1.14	2.80	−0.03	1.90	3.08

**Table 3:** Impact of model components on prediction performance.

Base	Map	Physical Constraint	Vehicle Feature	minADE_5_	minFDE_5_	KDE NLL_5_
✓	×	×	×	1.76	4.02	3.36
✓	✓	×	×	1.41	3.13	3.29
✓	×	✓	×	1.46	3.29	3.16
✓	×	×	✓	1.42	3.07	3.02
✓	✓	✓	×	1.29	2.90	3.03
✓	×	✓	✓	1.32	2.99	2.91
✓	✓	×	✓	1.37	3.04	3.23
✓	✓	✓	✓	**1.26**	**2.85**	**2.89**

**Figure A1:**
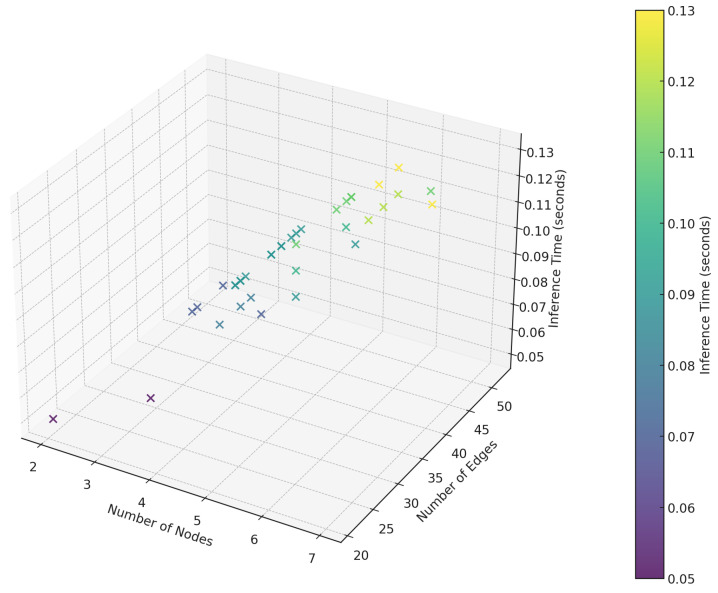
Three-dimensional scatter plot illustrating the relationship between inference time, the number of nodes, and the number of edges in online inference.

## Data Availability

The data used in this study are publicly available as part of the nuScenes dataset, which can be accessed at https://www.nuscenes.org.

## Abbreviations

The following abbreviations are used in this manuscript:

CVAE	Conditional Variational Autoencoder
RNN	Recurrent Neural Networks
GAN	Generative Adversarial Networks
GNN	Graph Neural Networks
LSTM	Long Short-Term Memory
MLP	Multi-Layer Perceptron
CNN	Convolutional Neural Networks
FC	Fully Connected (Layer)
GRU	Gated Recurrent Unit
GMM	Gaussian Mixture Model
minADE	Minimum Average Displacement Error
minFDE	Minimum Final Displacement Error
KDE NLL	Kernel Density Estimation Negative Log-Likelihood

## Appendix A. Online Runtime Performance

In the online inference process, we conducted a detailed analysis and measurement of the model’s runtime performance, covering inference time, frame rate (FPS), hardware environment, and memory usage. The experimental results indicate that the inference time and frame rate of the model on a CPU are closely related to the number of nodes and edges. An increase in the number of nodes and edges significantly increases the computational load, resulting in longer inference time and reduced frame rate.

During the experiment, the model was run on a CPU, with a model loading time of 0.03 s. The average inference time per timestep was approximately 0.09 s, corresponding to an average frame rate of 10.76 Hz. The inference time fluctuated with the number of nodes and edges, where a larger number of nodes and edges (e.g., 6–7 nodes and 40–50 edges) increased the inference time per timestep to 0.13 s, reducing the frame rate to around 7.7 Hz. Conversely, when there were fewer nodes and edges (e.g., 2–3 nodes and 20–30 edges), the inference time decreased to below 0.05 s, and the frame rate increased to 21 Hz or higher. This dynamic variation reflects the model’s computational complexity changing with input size. As shown in Figure A1, we use a 3D scatter plot to visually illustrate the relationship between inference time, the number of nodes, and the number of edges. Regarding memory usage, the CPU memory usage during online inference was approximately 25 GB.

Our approach can incrementally update new observation information without completely re-executing the forward pass. This is possible due to the use of an LSTM network structure, which allows new observational data to be directly input into the last LSTM unit of the encoder, effectively updating the model representation without repeating the entire inference process.

During inference, we chose to use a CPU to simulate the conditions of a real deployment environment. In many real-world applications, especially on resource-constrained edge devices, GPUs may not always be available. Additionally, for cost and energy efficiency, CPUs are often preferred. We tested inference on a CPU to ensure that the model maintains high real-time performance and stability under limited computational resources. This also demonstrates the model’s adaptability and potential applicability in low-resource environments.

The current CPU inference frame rate is 10–11 Hz, which meets the needs of some medium-level real-time applications, overall, the performance of online inference is as expected, and the model can adapt to changes in the number of nodes and edges in different scenarios.
